# Brain activity to transitional objects in patients with borderline personality disorder

**DOI:** 10.1038/s41598-017-13508-8

**Published:** 2017-10-13

**Authors:** Markus Kiefer, Ute Neff, Markus M. Schmid, Manfred Spitzer, Bernhard J. Connemann, Carlos Schönfeldt-Lecuona

**Affiliations:** Ulm University, Department of Psychiatry, Ulm, Germany

## Abstract

Adult patients with borderline personality disorders (BPD) frequently have attachments to inanimate transitional objects (TOs) such as stuffed animals. Using event-related potential (ERP) recordings, we determined in patients with BPD the neural correlates of the processing of these attachment-relevant objects and their functional significance. Sixteen female patients with BPD viewed pictures of their TOs, other familiar stuffed toys (familiar objects, FOs), and unfamiliar objects (UOs). ERPs in the patients were compared to those in 16 matched healthy controls who possessed a stuffed animal of comparably high familiarity. Here, we found a specific increase of frontal P3/LPP amplitude in patients with BPD, which was related to attachment anxiety and depression scores. Attachment-related TO stimuli in patients with BPD specifically modulated stages of emotional stimulus evaluation reflecting processing of self-relevance. The relation of the frontal ERP effect to patients’ attachment anxiety and depression highlights the function of TOs for coping with anxiety about being abandoned by significant others and for dealing with depressive symptoms.

## Introduction

Attachment to inanimate objects such as stuffed animals (transitional objects, TOs) is a frequent phenomenon in children and is part of their normal mental development^1^. While parents or other caregivers are children’s primary attachment figures^2^, TOs serve as complementary attachment objects reducing emotional distress, e.g., when separated from the parents or loved others^3–5^.

In some instances, the use of TOs continues into adulthood, possibly serving a similar auxiliary attachment function as in childhood^6^. Studies in hospital settings indicated that psychiatric^6^ or non-psychiatric asthma^7^ patients, who displayed stuffed animals or other TOs like jewelry or photographs at bedside, more likely have a personality disorder compared to the inpatient population in general. TO use was most pronounced in inpatients with borderline personality disorder (BPD)^6,8^. In a nonclinical community sample, people with attachments to TOs more likely met the criteria for a BPD diagnosis compared to others without TO use^9^. In patients with BPD, a TO might serve to cope with anxiety due to social rejection, with feelings of emptiness and might help reducing emotional lability^6,10,11^. Studies of TO use in patients with BPD mostly included women, presumably reflecting the fact that patients with BPD in hospital settings are predominantly female^6,11^. Due to the rare occurrence of male inpatients with BPD, gender differences in TO use have not been investigated.

As TO use is frequently observed in female adult patients with BPD, we aimed to know whether processing of these attachment-relevant objects is distinct from that of emotionally less salient objects in this patient population. Event-related potentials (ERPs) are useful to track the time course of attachment-related processing within the range of milliseconds^12^. The following ERP components are of theoretical interest in visual and emotional stimulus processing: The *P1* component, which is typically largest over the occipital scalp, indexes processing of visual stimulus features^13,14^. The *N1* or *N170* component, which is also largest at occipital electrodes is thought to reflect activation of visual object representations^15–17^. Activation of mnemonic representations such as those of familiar faces is associated with a larger *N250* amplitude over the occipito-temporal scalp^18,19^. In a similar latency range, emotional stimuli have shown to elicit a negative potential (early posterior negativity, *EPN*)^20^ at occipital electrodes, related to detection of emotional significance. Finally, the parietal *P3* or late positive potential (*LPP*) is thought to index stimulus evaluation^21,22^.

Even though TO use, predominantly in female patients with BPD, has been documented in various scientific studies as described above, the electrophysiological correlates of TO processing have not been investigated yet. However, ERP studies in healthy participants on the processing of attractive or attachment-relevant faces allow inferences concerning the stage at which attachment-relevant stimulus information associated with TOs is being processed. Evidence regarding the ERP latency range of the influences of attachment-related facial stimuli in healthy female and male participants is not homogenous, although the majority of studies suggest a modulation of later mnemonic, emotional or evaluative processing stages in both genders starting at about 200 ms after stimulus onset (EPN, N250, P3/LPP, for N1 effects, see refs^23,24^): Faces of the own child increased N250 amplitudes in fathers^25^. Attractive faces modulated a posterior negativity, presumably the EPN, in samples including male and female participants^24,26^. Finally, in an even later time range, P3 and LPP responded to the face of a romantic partner in women^27,28^, to the face of the own child in a mixed sample of both mothers and fathers^29^, and to attractive faces in a sample of both genders^24,26^. Furthermore, objects owned by the subject differed from familiar objects owned by others in the P3/LPP range at frontal electrodes in a mixed sample of women and men, suggesting that this effect indexes self-relevance^30^. As these earlier ERP studies in healthy populations included samples of both genders or samples of only one gender, gender differences in the neural correlates of attachment-relevant stimuli could not systematically be elucidated. However, the studies reviewed above did not suggest differences between women and men.

**Figure 1:**
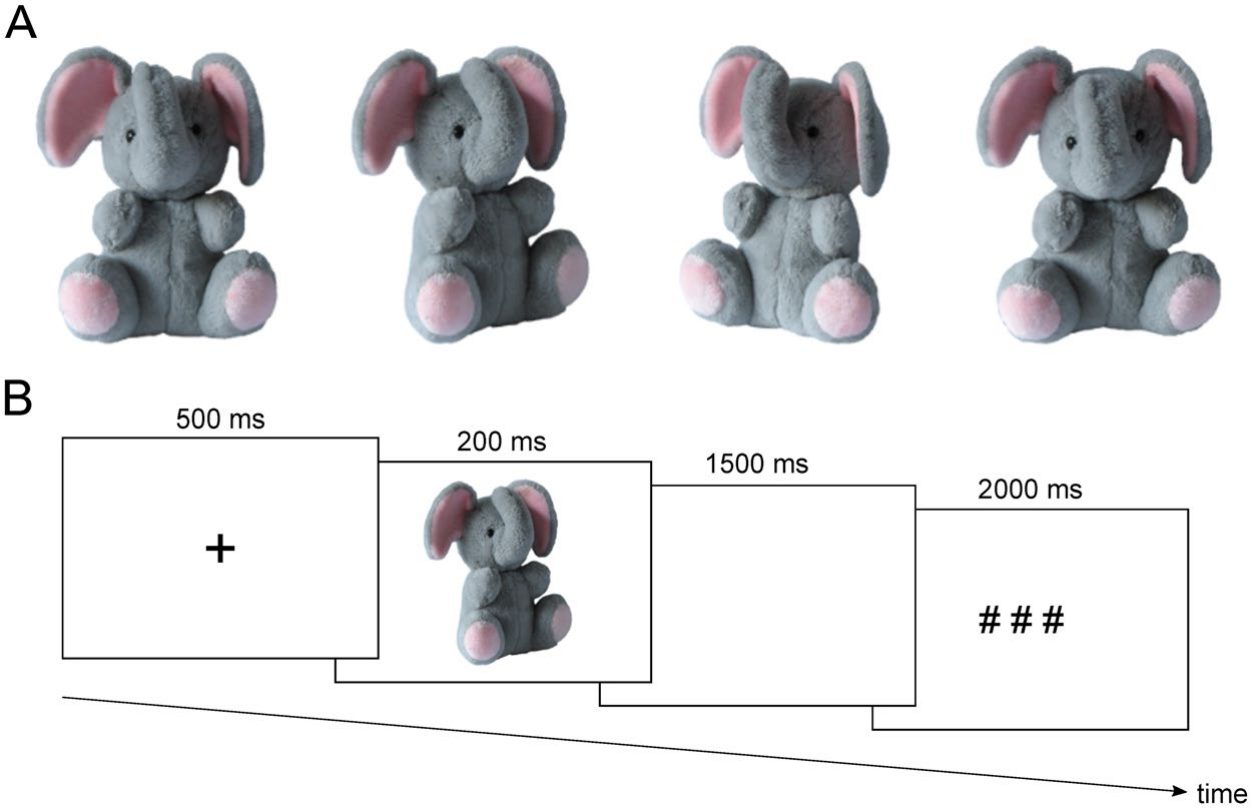
(**A**) Examples of the picture stimuli (shown is a stuffed animal in the possession of one of the authors). Pictures of the stuffed animals were taken from four different angles (front, 40° from left, 40° from right, 30° from top), in order to increase recognition difficulty in the 1-back task of the main experiment. (**B**) Sequence of events in one trial. Participants viewed a pseudo-randomized sequence of 288 pictures of the TO/HFO, FO and UO conditions within a 1-back task: They had to indicate by a button press with the right index finger, whether in the current trial the same object as in the previous trial was presented, irrespective of the angle the object pictures were taken. This 1-back task allowed us to record ERPs without the influence of a motor response in the critical non-repetition trials, while participants maintained attention to the stimulation stream. Abbreviation: ms = milliseconds.

As TOs display an exceptional attachment value for patients with BPD, in the present study we used recordings of electrical brain activity to assess, whether these patients process pictures of their TOs differently from pictures of familiar, but emotionally less salient objects (FOs), and unfamiliar objects (UOs). In particular, based on the known functional signficance of ERP components reflecting visual and emotional processing, we aimed at determining in patients with BPD the stage, at which attachment-relevant information of TOs is processed. Furthermore, we were interested in how these electrophysiological correlates of TO processing relate to clinical symptoms and attachment representations in this patient group. As patients with BPD in hospital settings are predominantly women^11^ and as we wanted to keep the sample homogenous, we only recruited female patients with BPD (Table 1) who possessed stuffed animals as TOs (Fig. 1A). Patients were compared with matched healthy female controls who possessed a favorite stuffed animal of comparably high familiarity (HFO), but without attachment function. Status of the object as TO or HFO was confirmed using the Transitional Object Questionnaire (TOQ)^8^. The comparison between FOs (i.e. moderately familiar, or liked, stuffed animals) and UOs served to estimate general familiarity effects on ERPs in both groups. Participants viewed a randomized sequence of TO/HFO, FO and UO pictures and indicated by a button press whether two subsequently presented stimuli showed the same stuffed animal (Fig. 1B). This task ensured attention to the stimuli.

**Table 1:** Demographic and clinical data of patients with BPD (PAT) and healthy comparison participants (COMP). Shown are means per condition, +/−95% confidence intervals (CI) and standard deviations (in brackets). L-P-S (Leistungsprüfsystem for evaluating non-verbal intelligence), standardized T scores; BDI-II: Beck-Depression-Inventory; HAM-D: Hamilton-Depression-Rating-Scale; AAS: Adult Attachment Scale.

	PAT n = 16	COMP n = 16	Statistics	
			t(30)	p
Age	29.3 (8.8) CI 24.8–34.0	29.0 (8.7) CI 24.4–33.6	0.13	0.90
Education	10.4 (1.8) CI 9.5–11.4	10.6 (1.6) CI 9.7–11.4	−0.20	0.84
L-P-S T scores	55.7 (5.4) CI 52.8–58.5	56.4 (9.5) CI 51.3–61.5	−0.27	0.79
Handedness (r/l)	14/2	14/2	n.a.	n.a.
BDI-II	33.8 (14.3) CI 26.1–41.4	2.9 (4.0) CI 0.8–5.0	8.30	>0.0001
HAM-D	11.7 (5.7) CI 8.7–14.7	0.6 (1.4) CI −0.2–1.3	7.62	>0.0001
AAS Close	14.8 (4.3) CI 12.5–17.1	26.1 (2.7) CI 24.7–27.6	−8.90	>0.0001
AAS Depend	16.3 (5.3) CI 13.4–19.1	25.6 (3.6) CI 23.7–27.5	−5.83	>0.0001
AAS Anxiety	20.4 (5.4) CI 17.5–23.3	10.1 (3.6) CI 8.3–12.1	6.24	>0.0001

We assumed that patients with BPD assign emotional valence to TOs due to their function as attachment figure, which is absent for HFOs in controls. Processing of HFOs, FOs and UOs in controls should therefore reflect only varying degrees of familiarity and valence, which would induce a graded modulation of the occipital N250 and EPN ERP components, with largest amplitudes for HFOs, followed by FOs and UOs. TOs in the patient group should elicit an enlarged EPN, thereby enhancing the posterior negativity to TOs compared with HFOs and FOs. Furthermore, as self-relevant objects previously elicited an enlarged P3/LPP amplitude at frontal electrodes^30^, we expected to find a similar effect to TOs in patients with BPD. The functional significance of the TO ERP effect for attachment processing should be confirmed by its correlation with patients’ attachment representations.

## Results

### Ratings of the stuffed animals

Repeated-measures analyses of variance (ANOVAs) with the within-subject factor category and the between-subject factor group revealed main effects of category for both familiarity *(F*(2,60) = 407.51, ε = 0.78, *p* < 0.0001, η_p_² = 0.931) and valence (*F*(2,60) = 137.49, ε = 0.88, *p* < 0.0001, η_p_² = 0.820), but no interactions (all Fs < 1.1, all ps > 0.31). Tukey HSD post-hoc tests yielded significant differences across all three stimulus categories (all ps < 0.001): TO/HFO stimuli were rated as more familiar and as emotionally more positive compared with FOs followed by UOs (Table 2).

**Table 2:** Behavioral data of patients with BPD and healthy comparison participants.

	UO		FO		TO/HFO	
	PAT	COMP	PAT	COMP	PAT	COMP
Familiarity	1.0 (0) CI n.a.	1.1 (0.3) CI 0.9–1.3	4.3 (1.1) CI 3.7–4.9	4.3 (1.0) CI 3.7–4.8	5.9 (0.3) CI 5.8–6.0	5.6 (0.9) CI 5.1–6.0
Valence	2.0 (1.2 CI 1.4–2.6)	2.0 (1.2) CI 1.4–2.6	4.0 (1.1) CI 3.4–4.6	4.0 (0.7) CI 3.6–4.4	5.8 (0.5) CI 5.5–6.1	5.6 (0.7) CI 5.3–5.8
RT (ms)	557 (151) CI 476–637	553 (150) CI 474–633	537 (130) CI 467–606	547 (166) CI 458–636	514 (99) CI 461–567	538 (173) CI 445–630
d′	3.3 (0.7) CI 3.0–3.7	3.8 (0.5) 3.6–4.1	3.3 (0.6) CI 3.0–3.6	3.8 (0.5) CI 3.5–4.1	3.8 (0.6) CI 3.5–4.1	3.7 (0.6) CI 3.4–4.0

Results of the post-experimental familiarity and valence ratings as well as reaction times (RT) in milliseconds (ms) and accuracy (d’) in the 1-back task of the main experiment as a function of stimulus category. Shown are means per condition, +/−95% confidence intervals (CI) and standard deviations (in brackets). UO: unfamiliar object; FO: familiar object; TO: transitional object; HFO: highly familiar object; PAT: patients with BPD; COMP: comparison participants.

### Performance in the 1-back task

Analysis of reaction times (RTs) in the stimulus repetition trials was based on mean RT of correct responses in each condition. Outlying responses (individual mean +/−2 SD) were excluded from RT analysis (3.9% of the entire data set). A repeated-measures ANOVA with the within-subject factor category and the between-subject factor group yielded a significant main effect of category (*F*(2,60) = 3.83, ε = 0.80, *p* = 0.036, η_p_² = 0.113), but Tukey HSD post-hoc tests did not reveal reliable differences between conditions. The interaction between category and group was not significant (F < 0.80, p > 0.45). Mean RT was 536 ms (SD = 123) in the patient group and 546 ms (SD = 160) in the control group (Table 2).

Accuracy in detecting stimulus repetitions was assessed by calculating d’ sensitivity measures from hit (correct responses to repetitions) and false alarm (erroneous responses to non-repetitions) rates^31^ (Table 2). A repeated-measures ANOVA revealed a significant interaction between the within-subject factor category and the between-subject factor group (*F*(2,60) = 6.25, ε = 0.93, *p* = 0.004, η_p_² = 0.172). According to Tukey HSD post-hoc tests, in the patient group d’ was significantly higher for TOs than for FOs (p = 0.01) or UOs (p = 0.03). Mean differences within the control group or between groups were not statistically reliable (all ps > 0.10).

### Electrophysiological results

**Figure 2:**
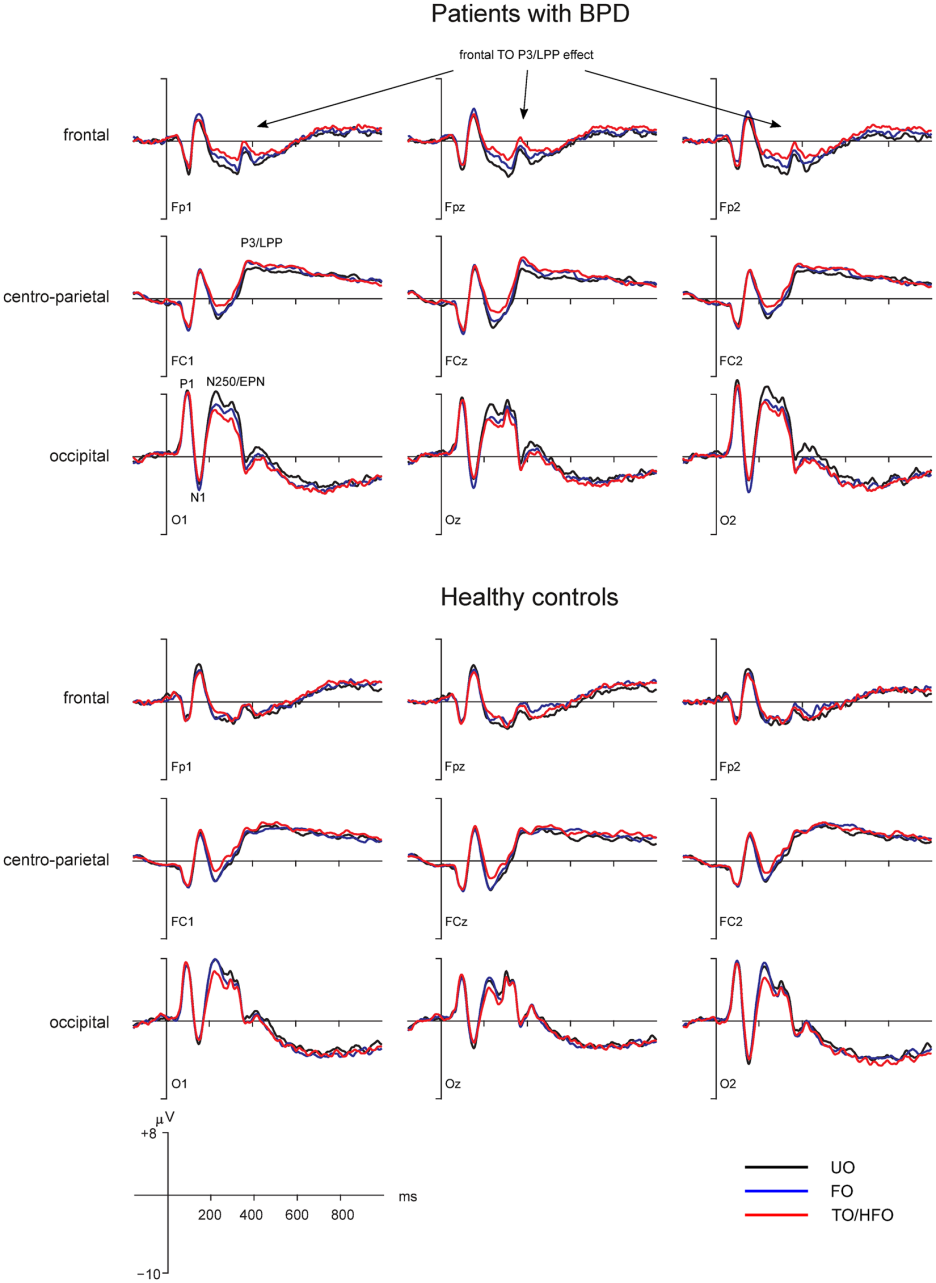
Grand-averaged ERPs to unfamiliar (UO), familiar (FO) and transitional object/highly familiar object (TO/HFO) stimuli in patients with BPD (n = 16) and healthy controls (n = 16). Shown are ERP plots of representative electrodes in occipital, centro-parietal and frontal scalp regions of interest. The y-axes indicates stimulus onset. Positive potentials are plotted upwards. Abbreviations: µV = microvolt; ms = milliseconds.

Based on the known topography of the P1, N1, N250/EPN and P3/LPP ERP components, which were of theoretical interest (see the introductory section), statistical analyses of ERPs were performed at bilateral and midline electrodes in occipital (P1, N1, N250/EPN), centro-parietal (P3/LPP) and frontal (frontal P3/LPP) scalp regions (Fig. 2). Mean amplitude of these ERP components was analyzed in time windows centered on their respective peaks (see also methods section) in the corresponding scalp region of interest. To reduce the complexity of the results section, we only describe effects of theoretical interest that include the factor stimulus category.

### P1 (94–114 ms) and N1 (144–168 ms)

For P1 and N1 amplitude, which was analyzed at occipital electrodes, effects of the factor category were not significant (all Fs < 1.32, p > 0.27).

### N250/EPN (220–320 ms)

Amplitude of the N250/EPN component over the occipital scalp was significantly different for the TO/HFO, FO and UO stimuli: The ANOVA revealed a main effect of the factor category, *F*(2,60) = 15.33, ε = 0.83, *p* < 0.0001, η_p_² = 0.338, while subsequent post-hoc tests revealed statistically reliable differences between all conditions (all ps < 0.03). As expected for an N250/EPN, ERPs to TOs/HFOs were most negative, followed by ERPs to FOs and UOs. Interactions between category and group were not significant (all Fs < 0.81, all ps > 0.43) suggesting that the N250/EPN pattern for the different stimulus categories was comparable for the patient and the healthy comparison group.

### P3/LPP (320–520 ms)

ERPs were analyzed over centro-parietal and frontal scalp regions in order to capture the classical cento-parietal and the frontal P3/LPP. For the cento-parietal P3/LPP, a main effect of the factor category was obtained (*F*(2,60) = 3.88, ε = 0.99, *p* = 0.026, η_p_² = 0.115). Post-hoc tests yielded more positive ERPs to TOs/HFOs compared with UOs (p = 0.022). The ERP differences between TOs/HFOs and FOs and between FOs und UOs were not statistically reliable (all ps > 0.16).

Most importantly, at frontal electrodes the interaction between category and group was significant *(F*(2,60) = 5.07, ε = 0.93, *p* = 0.011, η_p_² = 0.145). Post-hoc tests revealed differences between categories only in patients with BPD, but not in controls (all ps > 0.19). In patients, TOs elicited more positive ERPs than FOs and UOs (all ps < 0.03). ERPs to the latter two stimulus categories did not significantly differ from each other (p = 0.11). Hence, frontal ERPs in the P3/LPP time window were only modulated by TOs in the patient group (Fig. 2).

### Correlation analyses

**Figure 3:**
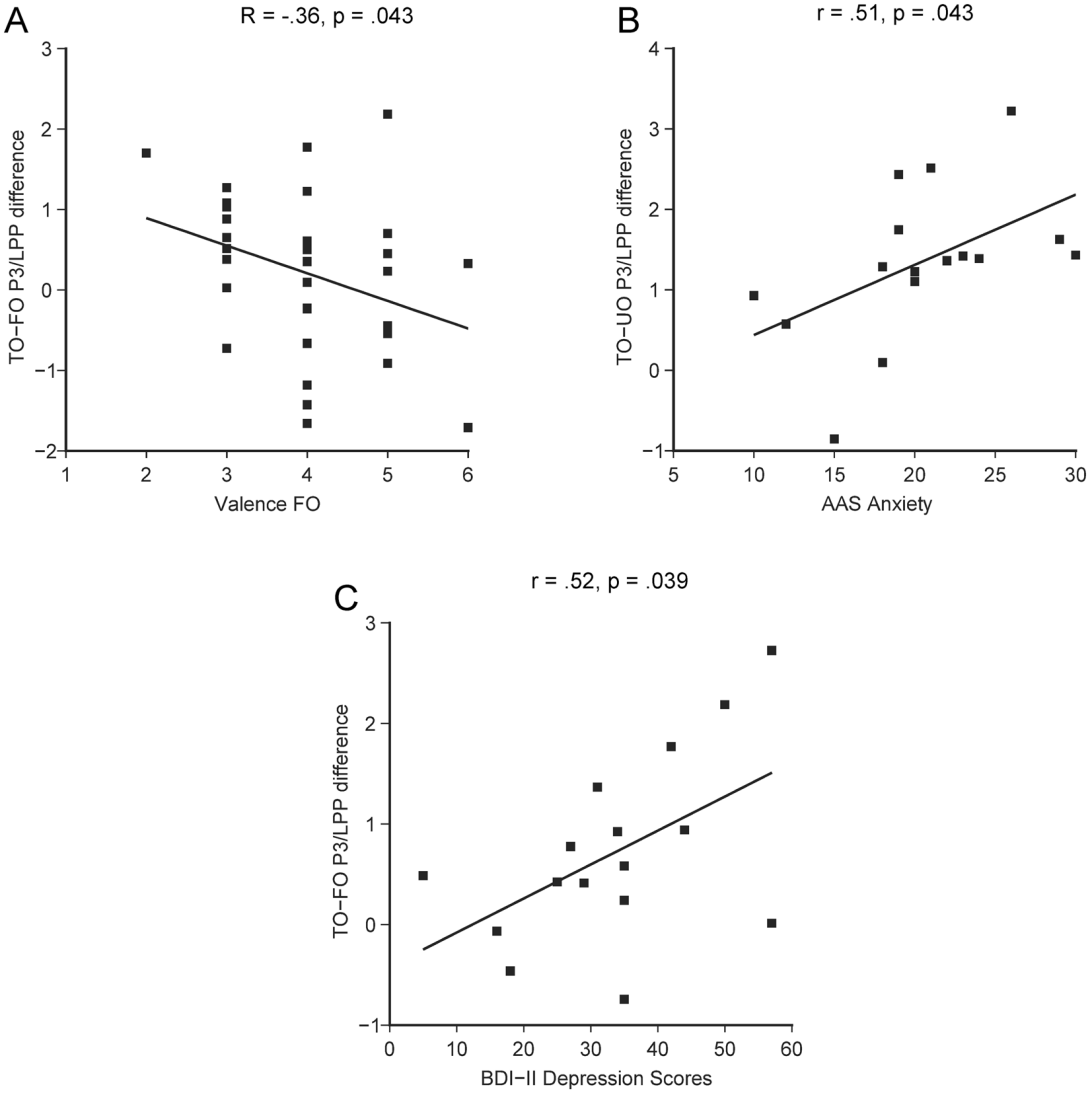
Bivariate scatterplots showing significant correlations with frontal P3/LPP ERP effects. (**A**) Correlation between right TO/HFO-FO ERP differences (in μV) and FO valence ratings in the entire sample of patients and controls (n = 32). (**B**) Correlation between right TO-UO ERP differences (in μV) and the AAS scale attachment anxiety in patients with BPD (n = 16). (**C**) Correlation between midline TO-FO ERP differences (in μV) and BDI-II depression scores in patients with BPD (n = 16).

To assess whether the frontal P3/LPP ERP effect to TOs was related to clinical variables of the patients as well as ratings of the stimuli, we performed correlation analyses based on the ERP differences TO/HFO-FO, TO/HFO-UO, separately for left, right and midline frontal electrodes. In the entire sample (patients and controls) we correlated valence ratings of FOs and UOs as well as familiarity ratings of FOs with TO/HFO ERP effects using Spearman rank correlation. Valence ratings of TOs/HFOs as well as familiarity ratings of TOs/HFOs and UOs were not entered into analysis, because they did not show substantial variation across participants. We found a significant negative association between valence ratings of FOs and the right frontal TO/HFO-FO ERP difference (R = −0.36, p = 0.043): A higher valence of FOs was associated with a reduced ERP difference between TOs/HFOs and FOs (Fig. 3A).

In the patient group, we related frontal P3/LPP TO ERP effects to depression scores of the Beck-Depression-Inventory (BDI-II) and of the Hamilton-Depression-Rating-Scale (HAM-D), Chlorpromazine equivalence dose of neuroleptic medication, Fluoxetine equivalence dose of antidepressant medication, and attachment (AAS) using product-moment correlations. Valence ratings of FOs and UOs as well as familiarity ratings of FOs were related to the TO ERP effects using Spearman rank correlations. There was a statistically reliable positive correlation of the AAS subscale Anxiety with the TO-UO ERP difference over the right hemisphere (r = 0.51, p = 0.043). Greater anxiety about being abandoned by significant others was related to an increased right frontal TO ERP effect (Fig. 3B). Furthermore, BDI-II scores showed a positive correlation with the TO-FO ERP difference over the frontal scalp (Fig. 3C), significantly over the frontal midline (r = 0.52, p = 0.039), non-significantly over the left and right frontal scalp (r = 0.41, p = 0.11, r = 0.42, p = 0.10): Similar to anxiety, larger BDI-II depression scores were associated with an increased frontal P3/LPP ERP effect to TOs. There was a trend towards a positive correlation of the equivalence dose of antidepressant medication with the TO-FO difference over the left hemisphere (r = 0.48, p = 0.058). Increased dose of antidepressants were related to an increased left frontal TO-FO ERP difference. Valence and familiarity of the FO tended to be correlated with the right TO-FO ERP difference (valence: R = −0.46, p = 0.07; familiarity: R = −0.43, p = 0.09): Higher valence and familiarity of the FO were associated with a decreased TO-FO ERP difference. Other correlations in the patient group were far from being statistically reliable (p > 0.12). In the control group, correlations of the frontal HFO-FO/UO ERP differences with BDI-II, HAM-D, AAS scales, valence and familiarity ratings were not statistically reliable (p > 0.22).

### ERP source analyses

**Figure 4:**
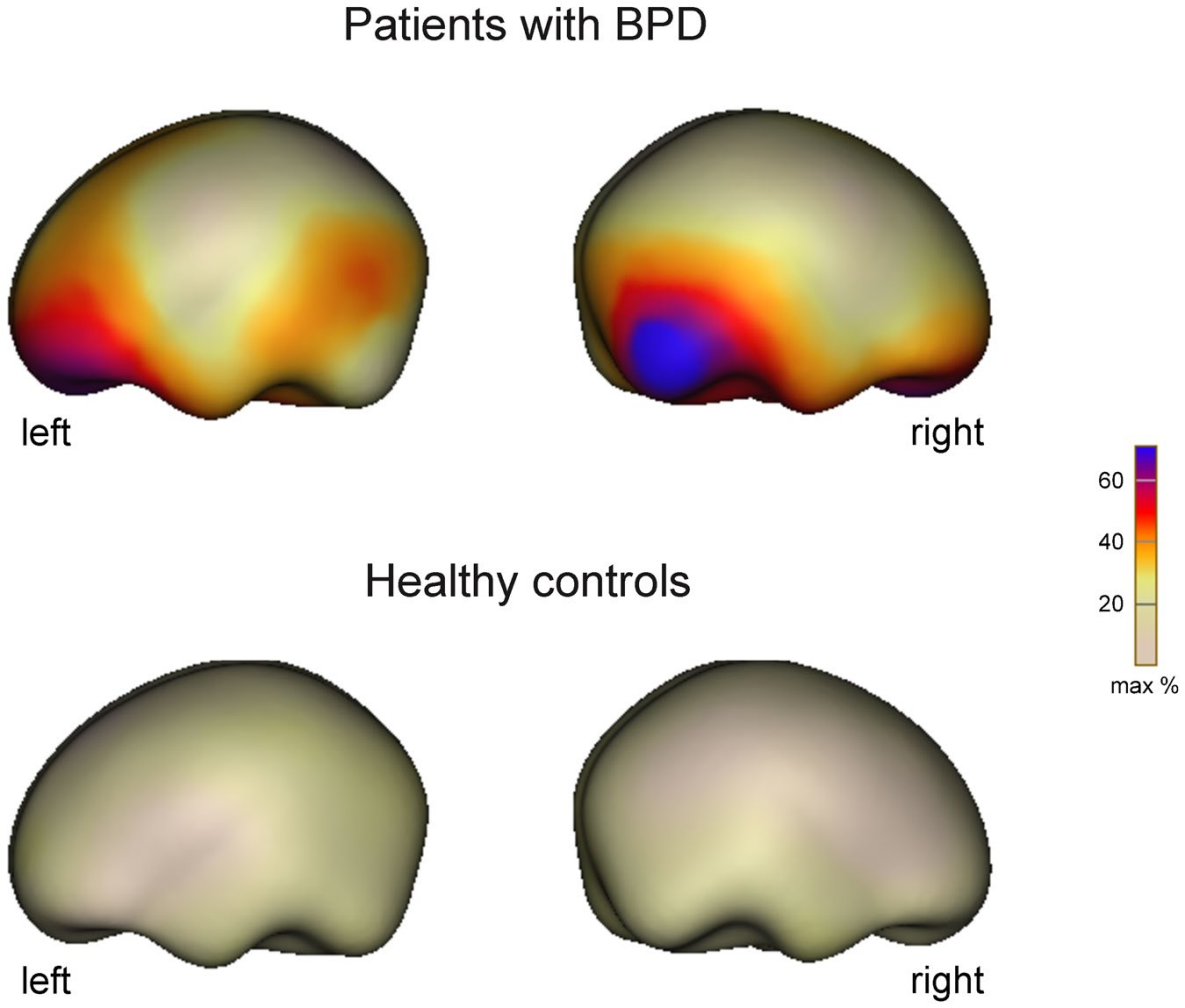
Neural source estimates of the TO P3/LPP effect in patients with BPD and healthy controls. Sources were calculated according to the minimum norm algorithm from the ERP difference wave TO-FO. Maps of cortical currents are shown at the maximum of global field power (338 ms after stimulus onset) displayed on a standard brain surface. Substantial source activity in bilateral occipito-temporal and frontal areas was only found in the patient, but not in the healthy comparison group.

Source analyses of the TO/HFO effect in the P3/LPP time window at the time point of the maximum of the scalp effects revealed substantial source activity only in the patient group (Fig. 4): Analysis suggested source activity in bilateral occipito-temporal areas as well as in frontal areas including orbito-frontal and prefrontal cortex (for source analyses of the N250/EPN effect, see the Supplementary Information).

## Discussion

The present ERP study investigated the time course of brain activity in response to the processing of attachment-relevant stuffed animals, TOs, in patients with BPD. We found in the patient group an increased frontal P3/LPP amplitude in response to TOs. The magnitude of the frontal TO ERP effect in the patient group was significantly associated with attachment anxiety and depression. ERP differences in the earlier P1 and N1 time windows were not statistically reliable suggesting that attachment-relevant information is accessed at later stimulus evaluation stages.

### ERP effects in the N250/EPN time window (220–320 ms)

In the N250/EPN time window, we found an occipital category ERP effect that was comparable across groups. There was only a graded difference between TOs/HFOs, FOs and UOs reflecting stimulus familiarity/valence. Contrary to our expectations, the status of TOs as attachment figures did not boost early assignment of emotional significance (see also^30^). As TOs/HFOs, FOs and UOs varied with regard to both valence and familiarity, this occipital ERP effect most likely reflects both a modulation of the N250 ERP component indexing activation of mnemonic representations^18,19^, and a modulation of the EPN reflecting emotional significance detection^20^. This assumption is also corroborated by source analyses of the scalp ERP effect in the N250/EPN time window (see the Supplementary Information), which revealed activity within bilateral occipito-temporal and inferior parietal areas. These generators in the ventral and dorsal visual streams^32^ are compatible with earlier findings from source analyses of both the N250/N250r^18,33^ and the EPN^34^.

### ERP effects in the P3/LPP time window (320–520 ms)

In line with our expectations and with earlier work^30^, the P3/LPP effect of stimulus category differed across groups at frontal electrodes: Only in patients with BPD, TOs elicited larger frontal P3/LPP amplitude than the other stimulus categories. These results converge with previous work in healthy participants demonstrating only frontal, but not parietal P3/LPP effects for personally owned objects^30^. This suggest that object self-relevance, which is most pronounced for patients’ TOs, is reflected by the frontal P3/LPP amplitude.

At centro-parietal electrodes, at which the P3/LPP is typically largest, only a larger amplitude for TO/HFO stimuli compared with UOs was found independently of the factor group. These occipital and centro-parietal effects in response to TOs/HFOs most likely reflect general stimulus evaluation processes^21,22^ based on the higher valence and/or familiarity of TOs/HFOs, which were comparable in either group.

The proposal that the frontal TO P3/LPP effect in the patient group reflects evaluation of emotional self-relevance due to the function of the TO as attachment figure is supported by the correlation analyses. The magnitude of the frontal TO effect was positively related to the attachment anxiety scores of the AAS (right frontal) and to BDI-II depression scores (most prominent midline frontal). This correlation pattern suggests that in patients with greater attachment anxiety and with more pronounced depressive symptoms emotional self-relevance of the TO is enhanced. This observation supports the notion that TOs serve in patients with BPD as supplementary attachment figure, which is used for emotion regulation, in particular for reducing emotional distress^6,10,11^. The right frontal P3/LPP difference between TOs and FOs was negatively related to the valence of the FO: The higher the valence of the FO was rated, the more similar was right frontal P3/LPP amplitude for these stimulus categories.

Attachment-related processing involves prefrontal and orbito-frontal as well as occipital areas in both hemispheres^35,36^. Nevertheless, neural circuits in the right prefrontal and orbito-frontal cortex might be more important for emotional evaluation of attachment relevant stimuli compared with homologue areas in the left hemisphere as found in earlier work^37–40^ explaining why correlations with attachment anxiety and valence were only significant for the right frontal ERP effect.

Although the localizational value of ERPs have to be viewed with caution^41^ and the present source analyses were only descriptive, minimum norm estimates of the scalp P3/LPP ERP effect to TOs suggest generators in orbito-frontal and prefrontal areas as well as in occipito-temporal regions, similarly to earlier neuroimaging results^35,36^. Activity in orbito-frontal and prefrontal areas most likely arises from evaluating the emotional significance of the stimulus^37–39^. Activity in occipital areas might index enhanced visual processing of the TO stimuli, possibly driven by their high emotional valence and familiarity^36^.

While attachment anxiety or depression were associated with the right or midline frontal P3/LPP TO effect, dose of antidepressant medication tended to be related to the left frontal P3/LPP TO-FO difference. This trend for a correlation between the left frontal P3/LPP TO effect and antidepressant medication might be due to factors associated with the prescription of higher doses (see also the Supplementary Information). Alternatively, a high dose of antidepressant medication might enhance functionality of prefrontal circuits similar to observations in patients with major depression^42–44^, thereby boosting the left frontal P3/LPP TO effect. These interpretations are clearly speculative and deserve further investigations. In any case, as antidepressant medication tended to correlate only with the left frontal P3/LPP TO effect, it can be ruled out that right and midline frontal P3/LPP differences between patients and controls were biased or even caused by antidepressant medication. Dose of antipsychotic medication was not related to the TO effect rendering an influence on the results unlikely.

As our sample comprised only women, it would be interesting to assess whether similar effects would be found in male patients with BPD. Further studies could therefore assess gender differences in the neural correlates of the processing of attachment-related objects. Furthermore, all patients were medicated, and the dose of antidepressant medication tended to modulate the left frontal P3/LPP TO effect, although the right and midline frontal P3/LPP TO effects were not affected by the dose. In order to further determine the influence of medication on the TO ERP effects, it would be desirable to investigate unmedicated patients in future studies. Finally, in this study we investigated the neural correlates of TO processing only in patients with BPD. Previously, we observed that patients with other psychiatric diagnoses (e.g., other personality disorders or major depression) also possess TOs, albeit less frequently than patients with BPD^6^. It must remain open, whether the frontal P3/LPP TO effect is specific to patients with BPD or would also be found in other patient groups.

## Conclusion

Our results demonstrate that viewing attachment-related TO stimuli in patients with BPD specifically modulated later stages of emotional stimulus evaluation as indexed by the P3/LPP. Earlier processing stages related to activation of mnemonic representations and to assignment of emotional value as indexed by the N250 and EPN components, respectively, were only modulated by general familiarity and valence of the stimuli, but not by the emotional status of TOs as attachment figure. The frontal TO effect was related to patients’ attachment anxiety and depression highlighting the function of TOs for coping with anxiety about being abandoned by significant others and for dealing with depressive symptoms

## Methods

### Participants

Sixteen healthy female comparison participants and sixteen medicated female patients with BPD contributed data to this study (Table 1). We investigated only female participants, because patients with BPD in hospital settings are predominantly female. Furthermore, the possession of stuffed animals and TOs, a prerequisite for participation in our study, is more frequent in women than in men. Data of additional five patients had to be excluded from analyses, four due to excessive artifacts in the EEG recordings and one due to premature abortion of the experimental session. Patients were inpatients or outpatients of the Department of Psychiatry and Psychotherapy III at Ulm University. All participants were right-handed^45^, with normal or corrected-to-normal vision without any signs of neurological illnesses according to a standardized in-house questionnaire. Inclusion criteria for all participants was the possession of stuffed animals, as TO/HFO and as FO. We restricted our study to participants with stuffed animals as potential TOs and excluded participants with other emotional objects (e.g., blankets), in order to minimize visual variability of the stimuli for the main experiment. Using the Transitional Object Questionnaire (TOQ^8^, we confirmed the status of a stuffed animal as TO in the participants of the patient group and ruled out this status in the comparison group (HFO/FO). Consistent with earlier work^6,8^, a stuffed animal was defined as TO, when it was categorized in the TO-Questionnaire as “quite important” or “essential”.

Psychiatric diagnosis of BPD was made by a psychiatrist of the hospital based on the structured clinical interview for axes II disorders of the DSM-IV (SCID-II)^46^. Additional psychiatric diagnoses were based on a structured interview according to DSM-IV criteria^47^. Patients with BPD had the following secondary diagnoses: recurrent depressive disorder (6), bulimia nervosa (2), post-traumatic stress disorder (2), obsessive-compulsive disorder (2), harmful alcohol use (2), combination of several personality disorders (3). Exclusion criterion was a severe depressive episode with psychotic symptoms and/or acute suicidality. All patients with BPD received antidepressants (12 SSRI, 3 SNRI, 1 NDRI) with a mean Fluoxetine equivalence dose of 42.2 mg/d, SD 16, range 20–81 mg/d (according to^48^). Thirteen patients additionally received antipsychotic medication with a Chlorpromazine equivalent dose of 252.9 mg/d, SD 254, range 25 mg/d – 800 mg/d (according to^49,50^), seven patients received anticonvulsant medication, two patients received benzodiazepines. Comparison participants were recruited by advertisements and were free from personal or family history of psychiatric disorders according to a standardized in-house questionnaire. Comparison participants were also screened for personality disorders using SCID-II. None of the comparison participants met the criteria of an axes II disorder. In all participants, depressive symptoms were assessed using the German Version^51^ of the Beck-Depression-Inventory (BDI-II) as self-rating scale^52^ and the Hamilton-Depression-Rating-Scale (HAM-D) as clinician rating scale^53^. Interviews for the HAM-D were performed by a trained psychiatrist. All participants also received the German version^54^ of the Adult Attachment Scale AAS^55^, in order to assess quality of attachment representations; AAS includes the subscales Close (acceptance of the closeness of others), Depend (feeling comfortable to rely on others), Anxiety (worrying about being abandoned by others). Non-verbal intelligence was measured with the L-P-S^56^. Participant groups did not differ significantly with regard to gender, age, years of education and non-verbal intelligence (Table 1). As expected, BPD patients showed significantly higher BDI-II and HAM-D scores. In comparison participants, depression scores in both scales (BDI-II and HAM-D) were low and below clinically relevant thresholds (scores <8). Patients with BPD also significantly differed from comparison participants on all AAS subscales: As expected, patients had lower Close and Depend scores, but higher Anxiety scores. All participants gave written informed consent. The procedures of the study have been approved by the Ethical Committee of Ulm University (No. 116/11). All experiments were performed in accordance with relevant guidelines and regulations.

### Material

For each participant, we took altogether eight color pictures of two stuffed animals in the possession of the participants (Fig. 1A). One animal meeting the criteria for a TO (patient group) or a HFO (comparison participants). In the patient group, the status of a stuffed animal as TO, i.e. emotionally significant attachment figure, was validated using the TOQ. In the comparison group, the HFO was defined as the most favorite stuffed animal that does not serve as attachment figure according to the TOQ. The second object serving as FO was defined as stuffed animal in the possession of the participants, which is less liked than the TO/HFO. For the UO control condition, we exchanged the TO/HFO pictures across participants within each group (e.g., patient 2 received as UO the TO of patient 1) so that TO/HFO and UO pictures were identical across participants within each group, thereby ruling out any ERP effects of visual object features such as form, texture or color. Pictures of the stuffed animals were taken from four different angles (front, 40° from left, 40° from right, 30° from top), in order to increase recognition difficulty. All pictures were taken under standardized lighting conditions against a white background on a day prior to the ERP experiment. The original background of the pictures was replaced by a uniform white background; brightness and contrast of the pictured objects was adjusted, when necessary. Size of the pictured objects was reduced to fit in a square of 280 × 280 pixels (256 colors).

### Procedure

Participants received the stimuli in the center of a cathode ray tube (CRT) computer screen against a white background synchronous with the screen refresh (refresh rate 16.67 ms) at a viewing distance of about 80 cm. Participants viewed a pseudo-randomized sequence of 288 pictures of the TO/HFO, FO and UO conditions within a 1-back task: They had to indicate by a button press with the right index finger, whether in the current trial the same object as in the previous trial was presented, irrespective of the angle the object pictures were taken. This 1-back task allowed us to record ERPs without the influence of a motor response in the critical non-repetition trials, while participants maintained attention to the stimulation stream. Unlike 2-back or 3-back tasks, 1-back tasks only require little working memory capacity^57^. There were 216 critical non-repetition trials (72 trials per condition; the four pictures taken from the different angles were presented 18 times) and 72 repetition trials (24 trials per condition; the four pictures taken from the different angles were presented six times). The 1-back repetition trials were discarded from further analyses. In the repetition trials, the viewpoint of the depicted object was always different from the viewpoint in the preceding trial so that accurate repetition detection depended on object recognition and not on simple matching of low-level visual features. Each trial started with a fixation cross (500 ms), followed by the object picture (200 ms) and a blank screen for 1500 ms, in which the response to the 1-back task was given. Thereafter, three hash marks (2000 ms) indicated the intertrial interval (Fig. 1B). The entire experiment was divided into five blocks of 57 or 58 trials. Prior to the main experiment, there were 15 practice trials, in which feedback about the correctness of the response was given, so that participants became familiarized with the task. Stimulus delivery and response recording was controlled by the software package Experimental Run Time System (Berisoft, Frankfurt, Germany). Participants were seated in an electrically shielded, sound attenuated booth in upright position and were instructed to blink only during the intertrial intervals. After the main experiment, participants received pictures of the individual TO/HFO, FO and UO on separate sheets of paper. They had to rate the familiarity (1: very unfamiliar; 6: very familiar) and the valence (1: very negative; 6 very positive) of the objects using a six-point rating scale. The entire experimental session including electrode set up, main experiment and post-experimental ratings lasted about two hours.

### Electrophysiological recordings and statistical analysis

Scalp voltages were continuously recorded (digitization rate = 500 Hz, band-pass = 0.001–100 Hz; BrainAmp, Brain Products, Gilching, Germany) using an equidistant montage of 64 Ag/AgCl electrodes mounted in an electrode cap (Easy Cap, Herrsching, Germany). Eye movements were recorded with supra- and infraorbital electrodes and with electrodes on the external canthi. Continuous electroencephalogram (EEG) was filtered (high cutoff: 30 Hz, 24 dB/octave attenuation; low cutoff: 0.1 Hz, 12 dB/octave attenuation, 50 Hz notch filter), segmented in relation to stimulus onset (−150–1000 ms) and baseline-corrected (−150–0 ms). Segments containing ocular artifacts or other artifacts in the recording channels were rejected. Artifact-free segments (ocular artifacts: amplitudes in VEOG and HEOG channels >+/− 40μV; other artifacts (amplitudes in recording channels’ >+/−70 μV) with correct responses were averaged separately for each experimental condition and electrode, and re-referenced to average reference^58,59^. Screening of individual ERPs did not reveal residual ocular activity in the data sets used for final analyses.

Statistical analyses of the ERP effects of category were based on mean voltages within the following time windows of interest: P1 (94–114 ms), N1 (144–168 ms), N250/EPN (220–320 ms) and P3/LPP (320–520 ms). Time windows were centered on the peak of the ERP components of theoretical interest in line with their known latency^60^. According to the known topography of these ERP components and based on the previous literature^24–26,30^, bilateral and midline electrodes in the following scalp regions were selected. P1, N1 and N250/EPN were analyzed at occipital electrodes (O1/O2, O9/O10, P7/P8, P9/P10, Iz, Oz). The classical P3/LPP was analyzed at centro-parietal electrodes (P1/P2, CP1/CP2, FC1/FC2, Pz, CPz, Cz), whereas the frontal P3/LPP related to stimulus self-relevance was assessed at frontal electrodes (Fp1/Fp2, AF3/AF4, F1/F2, Fpz, AFz, Fz). Statistical analysis involved repeated-measures analysis of variance (ANOVA) on mean voltages in the respective time windows with the within-subject factors category (TO/HFO, FO, UO) and laterality (left, midline, right) and the between-subject factor group (patients with BPD, healthy control participants). Violations of the sphericity assumption of the repeated-measures ANOVA were corrected using the Greenhouse-Geisser correction. The original degrees of freedom are reported together with the Greenhouse-Geisser ε and the corrected p-value. Significant main effects or interactions involving the factor category were further evaluated using Tukey HSD post-hoc tests. The significance level of all analyses was p < 0.05.

In order to estimate, which brain regions were implicated in the TO/HFO scalp ERP effects, we submitted the TO/HFO-FO ERP difference of significant effects to ERP source analyses using minimum norm estimation (minimum L2 norm, standardized realistic head model)^61^ implemented in BESA 6.1 (MEGIS, Gräfeling, Germany). We analyzed the difference of TOs/HFOs compared with FOs, to capture the TO effect in relation to a familiar, but emotionally less salient object. Sources were computed for the grand-averaged ERP difference waves between TO/HFO and FO stimuli to eliminate unspecific brain activity associated with stimulus processing. Cortical currents were determined at the time point of maximum effect size in the ERP difference waves to ensure optimal signal-to-noise-ratio^62^.

### Data availability

The data that support the findings of this study are available from the corresponding author upon reasonable request.

## Electronic supplementary material

Supplementary Information
