# Supplementary material for: Brain activity to transitional objects in patients with borderline personality disorder
Source: Sci Rep. 2017 Oct 13;7:13121. doi: 10.1038/s41598-017-13508-8 (PMC5640597; doi:10.1038/s41598-017-13508-8)
Supplement: Supplementary file 1 — Supplementary Information [file 41598_2017_13508_MOESM1_ESM.pdf]

Supplementary Information

Brain activity to transitional objects in patients with borderline personality disorder

Markus Kiefer, Ute Neff, Markus M. Schmid, Manfred Spitzer, Bernhard J. Connemann, and Carlos Schönfeldt-Lecuona

Ulm University, Department of Psychiatry

Supplementary Results

ERP source analyses

In the N250/EPN time window, scalp ERP effects to TOs were related to source activity within bilateral occipito-temporal and inferior parietal areas in both patients and comparison participants (Supplementary Figure 1). In comparison participants activity in this brain network was strongly right lateralized, whereas in the patients activity was largely bilateral and extended along the inferior temporal cortex to the temporal pole.

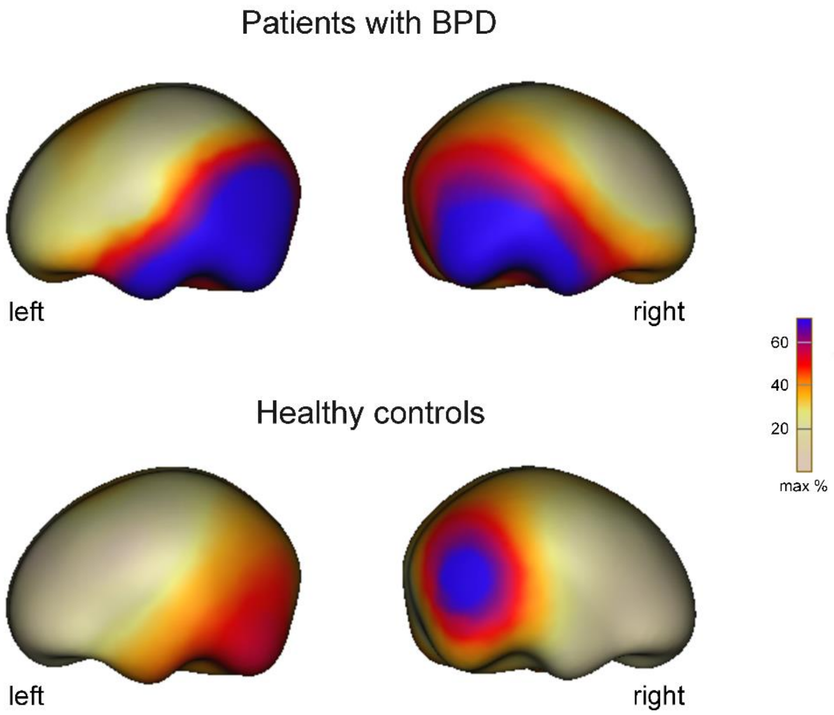

Supplementary Figure 1: Neural source estimates of the TO N250/EPN effect in patients with BPD and healthy controls. Sources were calculated according to the minimum norm algorithm from the ERP difference wave TO-FO. Maps of cortical currents are shown at the maximum of global field power (236 ms after stimulus onset).

Supplementary Discussion

ERP effects in the N250/EPN time window (220-320 ms)

The occipital category ERP effect in the N250/EPN time window was comparable across groups. This suggests that, contrary to our expectations, the status of TOs as attachment figure in patients with BPD did not boost their processing in this time range by enhancing their emotional significance. As TOs/HFOs, FOs and UOs vary with regard to both valence and familiarity, it is difficult to decide whether this occipital ERP effect is a modulation of the N250 ERP component indexing activation of mnemonic representations [1,2], or a modulation of the EPN reflecting emotional significance detection [3]. There was a gradient of both familiarity and valence for the different categories with TO/HFOs, rated as most familiar and as most positive, followed by FOs and UO. When calculating Spearman rank correlations for familiarity and valence ratings with the ERP differences between categories at left, right and midline occipital electrodes, there were significant positive correlations for both valence and familiarity of the FO with the TO/HFO-FO ERP difference at left (valence: $R = .40$, $p = .02$; familiarity: $R = .38$, $p = .03$) and midline electrodes (valence: $R = .43$, $p = .01$; familiarity: $R = .39$, $p = .03$). These positive correlations indicate that the TO/HFO-FO ERP difference, which has a negative polarity, is smaller the higher familiarity and valence of the FO was rated. Hence, when the FO was more familiar and more liked, the occipital ERP difference between TOs/HFOs and FOs was reduced, i.e. neural processing of TOs/HFOs and FOs became more similar. Possibly, the ERP effect of category at occipital electrodes includes a modulation of both the N250 and the EPN. Source analyses of the scalp ERP effect in the N250/EPN time window revealed activity within bilateral occipito-temporal and inferior parietal areas indexing activation of the ventral and dorsal visual streams [4]. Although the lateralization and extend of activity in the ventral stream seem to vary between patients and controls, these group differences are difficult to interpret because the present source analyses is only descriptive. Overall, these generators are compatible with earlier findings from source analyses of both the N250/N250r and the EPN. The N250/N250r has been referred to activity in the inferior temporal cortex [1,5]. The EPN has been related to activity within occipital, parietal regions and posterior limbic regions [6]. Thus, ocipito-temporal and parietal activity suggest to underlie the present TO/HFO ERP effect in the

N250/EPN time window seems to include brain areas also involved in the generation of both the N250 and the EPN. This also indicates that the TO/HFO ERP effect in the N250/EPN time window is probably due to a conjoint modulation of the N250 and EPN ERP components. In line with earlier findings with regard to the processing of a personally owned own vs. an object owned by others, the self-relevance of an object did not modulate ERP components within the time range of the N250 or EPN.

ERP effects in the P3/LPP time window (320-520 ms)

While attachment anxiety or depression were associated with the right frontal P3/LPP TO effect, dose of antidepressant medication tended to be related to the magnitude of the left frontal P3/LPP TO-FO difference, albeit non-significantly. This trend for a correlation between left frontal P3/LPP TO effect and antidepressant medication might be due to factors associated with the prescription of higher doses. Although dose of antidepressant medication did not significantly correlate with depressive symptoms or attachment scores, there was a trend towards a negative correlation with the Close scale of the AAS ($r = -.46$, $p = .07$, other ps > .30). Possibly, a reduced acceptance of closeness to others, which was correlated with high doses of antidepressant medication, might result in a higher emotional significance of the TO as attachment figure, which, in turn, yields larger higher frontal P3/LPP TO effect over the left hemisphere. Alternatively, a high dose of antidepressant medication might enhance functionality of prefrontal circuits similar to observations in patients with major depression [7-9], thereby boosting the left frontal P3/LPP TO effect. These interpretations are clearly speculative and deserve further investigations. In any case, as medication tended to correlate only with the left frontal P3/LPP TO effect, it can be ruled out that right frontal P3/LPP differences between patients and controls were biased or even caused by antidepressant medication.

Supplementary References

1. Schweinberger, S. R., Pickering, E. C., Jentzsch, I., Burton, A. M. & Kaufmann, J. M. Event-related brain potential evidence for a response of inferior temporal cortex to familiar face repetitions. *Cognit. Brain Res.* **14**, 398-409 (2002).

2. Tanaka, J. W., Curran, T., Porterfield, A. L. & Collins, D. Activation of preexisting and acquired face representations: The N250 event-related potential as an index of face familiarity. *J. Cognit. Neurosci.* **18**, 1488-1497 (2006).
3. Schupp, H. T. et al. The facilitated processing of threatening faces: An ERP analysis. *Emotion* **4**, 189-200 (2004).
4. Ungerleider, L. G. & Mishkin, M. in *Analysis of visual behavior* (eds. Ingle, D. J., Goodale, M. A. & Mansfield, R. J. W.) 549–586 (MIT Press, Cambridge, MA, 1982).
5. Williams, L. M., Palmer, D., Liddell, B. J., Song, L. & Gordon, E. The 'when' and 'where' of perceiving signals of threat versus non-threat. *Neuroimage* **31**, 458-467 (2006).
6. Keuper, K. et al. How 'love' and 'hate' differ from 'sleep': Using combined electro/magnetoencephalographic data to reveal the sources of early cortical responses to emotional words. *Hum. Brain Mapp.* **35**, 875-888 (2014).
7. Fitzgerald, P. B., Laird, A. R., Maller, J. & Daskalakis, Z. J. A meta-analytic study of changes in brain activation in depression. *Hum. Brain Mapp.* **29**, 683-695 (2008).
8. Delaveau, P. et al. Brain effects of antidepressants in major depression: A meta-analysis of emotional processing studies. *J. Affect. Disord.* **130**, 66-74 (2011).
9. Kennedy, S. H. et al. Changes in regional brain glucose metabolism measured with positron emission tomography after paroxetine treatment of major depression. *Am. J. Psychiat.* **158**, 899-905 (2001).
